# Preparation of g-C_3_N_4_/TNTs/CNTs Photocatalytic Composite Powder and Its Enhancement of Antifouling Performance of Polydimethylsiloxane Coatings

**DOI:** 10.3390/nano12142442

**Published:** 2022-07-16

**Authors:** Gang Xiong, Zhanping Zhang, Yuhong Qi

**Affiliations:** 1 Key Laboratory of Ship-Machinery Maintenance & Manufacture, Dalian Maritime University, Dalian 116000, China; xgg@dlmu.edu.cn (G.X.); yuhong_qi@dlmu.edu.cn (Y.Q.); 2 Department of Materials Science and Engineering, Dalian Maritime University, Dalian 116000, China

**Keywords:** graphitic carbon nitride, titanium dioxide nanotubes, carbon nanotubes, photocatalysis, marine antifouling coatings, polydimethylsiloxane

## Abstract

Semiconductor photocatalytic materials have shown potential in the field of antifouling due to their good antibacterial properties, stability, and nontoxic properties. It is an effective way to use them to improve the static antifouling performance of silicone antifouling coatings. g-C_3_N_4_/TNTs/CNTs (CNTC) photocatalytic composite powders were prepared and introduced into polydimethylsiloxane (PDMS) coatings to enhance their antifouling performance. Firstly, g-C_3_N_4_/TNTs with heterostructure were thermally polymerized by urea and TiO_2_ nanotubes (TNTs), and then g-C_3_N_4_/TNTs and multi-walled carbon nanotubes (CNTs) were composited to obtain CNTC. Finally, CNTC was added into PDMS to prepare g-C_3_N_4_/TNTs/CNTs/PDMS (CNTC/P) composite antifouling coating. The results showed that CNTC successfully recombined and formed a heterostructure, and the recombination rate of photogenerated carriers decreased after recombination. The addition of CNTC to PDMS increased the hydrophobicity and roughness while reducing the surface energy (SE) of the coatings. CNTC could effectively improve the anti-attachment performance of PDMS coatings to bacteria and benthic diatom. The bacterial attachment rate (A_B_) and benthic diatom attachment rate (A_D_) of CNTC/P-20 were, respectively, 13.1% and 63.1%; they are much lower than that of the coating without photocatalytic composite powder. This coating design provides a new idea for developing new “efficient” and “green” photocatalytic composite antifouling coatings.

## 1. Introduction

Marine ships and industrial facilities have long suffered from fouling organisms. Many fouling organisms adhere to the surface of vessels and industrial facilities, which will cause severe surface damage, reduce the service life, reduce their operational reliability, etc. [1,2]. At the same time, it also brings severe economic losses and environmental pollution. Applying antifouling coatings to the surfaces of ships and industrial facilities is an effective way to solve fouling biofouling. Currently, commercial coatings are usually antifouling agents containing copper compounds. Although the problem of fouling biofouling is solved, it also brings more or less environmental pollution [3,4]. Therefore, it is particularly crucial to develop antifouling coatings with high-efficiency antifouling properties and environmental friendliness. The fouling release antifouling coatings are environmentally friendly that not only can solve the problem of environmental pollution but also have excellent antifouling properties [5]. Foul release antifouling coatings include organofluorine and silicone polymers. Polydimethylsiloxane (PDMS) has superior fouling release properties to fluoropolymers [6,7]. Therefore, PDMS coatings are more promising. However, PDMS coatings are easily attached by fouling organisms under static conditions, and the surface is easily damaged by an external force. Usually, amphoteric polymers, antifouling agents, micro or nanopowders, and photocatalytic materials can be introduced to enhance the antifouling performance of PDMS coatings under static conditions [8,9]. Among them, nano-powder with photocatalytic activity can effectively improve the performance of PDMS coatings. Photocatalytic nanocomposite powders can enhance the antifouling properties, mechanical properties, and electrical and optical properties of PDMS coatings [6].

In recent years, photocatalytic materials have shown unique charm in the field of antifouling. Photocatalytic materials undergo photocatalytic reactions to generate a series of reactive oxygen species (ROS) under bandgap-matched illumination [10,11]. A variety of bacteria, proteins, and organic pollutants are inactivated or destroyed under the action of ROS [12]. Photocatalytic antifouling has attracted the attention of researchers all over the world because of its excellent antifouling efficiency, no harmful substances generated during the reaction process, and no pollution to the environment [13]. The performance of photocatalytic materials is closely related to their morphology, structure, surface properties, and specific surface area [14]. Furthermore, designing composite hetero nanostructures to facilitate the separation of photogenerated electron-hole pairs is an effective way to enhance photocatalytic activity. Among the numerous photocatalytic materials, graphitic carbon nitride (g-C_3_N_4_) has been widely studied due to its advantages of visible light response, simple preparation method, high stability, and environmental friendliness. However, the low specific surface area, limited active sites, and high recombination rate of photogenerated electron-hole pairs of pristine g-C_3_N_4_ also significantly limit the performance [15]. Common methods to improve the performance of g-C_3_N_4_ include element doping, optimizing the morphology, and constructing heterostructures [16,17,18]. Constructing heterostructures with suitable band structures is a condition for enhancing photocatalytic activity. Due to the unique energy band structure of TiO_2_, it can effectively promote the separation of electron-hole pairs. The tubular structure of TiO_2_ nanotubes has a larger specific surface area, which is conducive to the effective contact between g-C_3_N_4_ and TiO_2_, further enhancing the photocatalytic activity. Therefore, g-C_3_N_4_ is composited with TNTs to construct heterostructures to improve photocatalytic activity [19]. Xu et al. used this composite photocatalytic material for antibacterial coatings by grafting a layer of g-C_3_N_4_ on TNTs arrays, which significantly improved the bactericidal properties of the coatings [18]. Fu et al. prepared g-C_3_N_4_/Gr-CNTs/TiO_2_ Z-scheme photocatalysts by self-assembly using g-C_3_N_4_, TNTs, graphene (Gr), and CNTs, in which CNTs could effectively promote photogenerated charge carrier transfer and provide more surfaces for photocatalytic reactions [20]. Studies have shown that the addition of CNTs to PDMS can effectively improve its mechanical and hydrophobic properties while reducing the adhesion of fouling organisms [21,22,23,24]. In addition, CNTs also have good electrical conductivity and are suitable electron acceptors [25]. The composite formation of heterostructures based on g-C_3_N_4_ and TNTs can enhance their photocatalytic activity under visible light. As well, CNTs are advantageous in electrical conductivity and facilitated photogenerated carrier transfer. Inspired by this, we first prepared g-C_3_N_4_/TNTs/CNTs (CNTC) composite powder. We then introduced it into the PDMS antifouling coating to prepare a g-C_3_N_4_/TNTs/CNTs/PDMS (CNTC/P) composite antifouling coating to enhance the antifouling performance. A new composite powder was designed, which can absorb visible light for photocatalytic reaction. The heterostructure formed between them could promote the separation and transfer of charges so that the coating had better photocatalytic sterilization performance. The effect of the composite powder on the antifouling performance was evaluated by bacterial attachment experiments and benthic diatom attachment experiments. In addition, it was confirmed that the composite powder could effectively improve the antifouling performance of PDMS. This work provides a new idea for the application of photocatalytic materials in silicone antifouling coatings.

## 2. Experiment

### 2.1. Materials

The precursor for the preparation of graphitic carbon nitride (g-C_3_N_4_) was urea (AR), produced by Tianjin Fuchen Chemical Reagent Co., Ltd. (Tianjin, China). Titanium dioxide nanotubes (TNTs) were purchased from Xi’an Qiyue Biological Co., Ltd. (Xi’an, China). TNTs have an outer diameter of 10–15 nm and a length greater than 1 μm. Multiwalled carbon nanotubes (CNTs) were selected from Chengdu Zhongke Times Nanomaterials Co., Ltd. (Chengdu, China), with an outer diameter of 10–20 nm and a length of 0.5–2 μm. Isopropyl Alcohol (IPA, AR), Ethyl Orthosilicate (TEOS, AR), Xylene (AR), and Methyl Isobutyl Ketone (MIBK, AR) were purchased from Kermel Chemical Reagent Co., Ltd. (Tianjin, China). The catalyst bismuth neodecanoate (DY-20) was purchased from Shanghai Deyin Chemical Co., Ltd. (Shanghai, China). The film-forming substance used to prepare the coating was polydimethylsiloxane (PDMS, molecular weight 10,000), and its manufacturer is Dayi Chemical Co., Ltd. (Shandong, China). The coating fillers are fumed silica (AS-150), barium sulfate (AR), and heavy calcium (CMS-1000), which are produced by Zehui Chemical Co. (Guangdong, China), Ltd., Tianjin Damao Chemical Reagent Factory, and Heshan Chemical Co., Ltd. (Tianjin, China) respectively.

### 2.2. Preparation of g-C_3_N_4_/TNTs/CNTs (CNTC) Composite Powders

In the first step, urea and TNTs were ground and mixed uniformly at mass ratios of 10:1, 20:1, and 30:1, respectively. The mixed powder was then put into a crucible with a capacity of 50 mL, sealed with aluminum foil, and covered with a lid. Then, the crucible was put into an intelligent box-type high-temperature furnace, heated to 550 °C at 2 °C/min, and kept for 4 h. After the furnace temperature was naturally cooled to room temperature, g-C_3_N_4_/TNTs were obtained. In the second step, three kinds of g-C_3_N_4_/TNTs (2 g) and CNTs (0.5 g) were added to 200 mL of isopropanol (IPA) respectively and ultrasonicated for 1 h at room temperature. Then the mixture was stirred with a magnetic stirrer at room temperature for 10 h and dried to obtain g-C_3_N_4_/TNTs/CNTs (CNTC). According to the different mass ratios, the three composite powders obtained were denoted as CNTC-10, CNTC-20, and CNTC-30. In addition, g-C_3_N_4_/CNTs (CNC) were prepared, in which the mass ratio of g-C_3_N_4_ to CNTs was 2:1. The preparation method was the same as CNTC while TNTs were not added.

### 2.3. Preparation of Composite Coatings

In the first step, PDMA (50 g) was added to the sand mill dispersion mixing multi-purpose machine for medium and low-speed mixing. Barium sulfate (5 g), heavy calcium (19 g), fumed silica (3 g), auxiliary (3 g), and xylene (10 g) were added in sequence and the mixture was stirred at 3000 r/min for 30 min. The pretreated paint was then sanded with an industrial sander. The pretreated paint was into the sand mill, disperse and stir a multi-purpose machine, and stirred at 2000 r/min. Next, the mixture xylene (25 g) was added and was kept stirred evenly followed by adding CNTC or CNC (2 g). Subsequently, the rotational speed of the mixer was set to 4000 r/min, and the coating component A was obtained after stirring for 25 min. In the second step, component B was prepared by using xylene and ethyl orthosilicate in a mass ratio of 3:1, and component C was prepared by using methyl isobutyl ketone and DY-20 in a mass ratio of 7:3. In the third step, the A component and the B component were mixed and stirred evenly, and then the C component was added. Then the g-C_3_N_4_/TNTs/CNTs/PDMS (CNTC/P) composite antifouling coating was obtained after fully stirring. The components A, B, and C were mixed and stirred in a ratio of 10:2:1. To facilitate the test, CNTC/P was coated on a glass slide with a size of 75 mm × 25 mm × 1 mm and then injected into a polytetrafluoroethylene mold of 150 mm × 150 mm × 5 mm, cured at room temperature for 24 h. Finally, CNTC/P-10, CNTC/P-20, CNTC/P-30, and CNC/P were obtained. A blank control coating without CNTC and CNC was denoted as P using the same preparation method.

### 2.4. Characterization

#### 2.4.1. Crystal Structure

The crystal structure of the composite powder was measured using a D-max-Ultima X-ray diffractometer (XRD). The 2θ range used in the test was 10–80° and the scanning speed was 5°/min. The copper target used in the test equipment had a working voltage and current of 40 kV and 40 mA, respectively.

#### 2.4.2. Micromorphology

The microstructure of CNTC was measured by SUPRA 55 scanning electron microscope (SEM) and JEM-2100 super-resolution transmission electron microscope (TEM). Before the SEM test, the samples were ultrasonically dispersed in anhydrous ethanol and fixed on the conductive adhesive after drying. The operating voltage of the SEM is 3 kV. Before the TEM test, the composite powder was ultrasonically dispersed in ethanol and then a small amount of the dispersed liquid was placed on a copper mesh to dry naturally. The operating voltage of the transmission electron microscope was 200 kV. To observe the elemental distribution of the composite powder, we used Oxford Ultim Max type energy dispersive X-ray spectrometer (EDS) for testing.

#### 2.4.3. Surface Elemental Composition and Chemical State

The elemental composition and chemical bonds of CNTC were tested using the US-Thermo Fisher K-Alpha type X-ray photoelectron spectroscopy (XPS). The XPS was equipped with a monochromatic Al Kα X-ray source (1486.6 eV) operating at 100 W. Samples were analyzed under vacuum (*p* < 10^−8^ mbar) with a pass energy of 150 eV (measurement scan) or 50 eV (high-resolution scan). For adventitious carbon, all peaks would be calibrated using the C1s peak binding energy of 284.6 eV.

#### 2.4.4. Emission Spectra

The photoluminescence spectrum (PL) of CNTC was tested under an excitation light source with a wavelength of 365 nm using an F4600 spectrophotometer. Then, its photogenerated carrier recombination rate was characterized by the luminescence intensity.

#### 2.4.5. Contact Angle and Surface Energy

The contact angle of the coating was measured by a JC2000C contact angle meter, and its surface energy (SE) was calculated. The water contact angle (WCA) and the diiodomethane (CH_2_I_2_) contact angle (DCA) were tested, respectively. For each sample, five tests were performed at different locations. The images recorded by the software were saved, and the contact angles were measured using the software. SE was calculated from the measured data using the two-liquid method [26].

#### 2.4.6. Surface Morphology

The surface morphology of the coating was observed by the OLS4000 confocal laser microscope (CLSM), and the surface roughness was measured by LEXT software.

#### 2.4.7. Antifouling Properties

The marine bacterial adhesion experiment was carried out on the coating to test the anti-marine bacterial adhesion performance. The strains used in the experiments were obtained from the seawater of the Yellow Sea in Dalian (Dalian, China). The materials required for the experiment were sterilized using an autoclave. Three glass slide samples were selected for each coating for bacterial attachment experiments. In the first step, the samples were immersed in fresh seawater (the time for taking the seawater out of the sea was 2 h) and placed in a light incubator for 24 h (the light and dark time accounted for 12 h respectively). In the second step, the soaked sample was gently rinsed in 50 mL of sterilized seawater. After rinsing, the bacteria attached to the sample were transferred to sterilized seawater to dilute 10^−6^ times. In the third step, 10 μL of the diluted bacterial liquid was transferred to the medium and spread on the surface using a spreader. Finally, the medium was placed in a biochemical incubator with a temperature of 25 °C for 48 h. Then, it was taken out for counting and photographing to record the bacterial growth. To compare the antifouling performance more intuitively, the ratio of the number of colonies of CNTC/P and CNC/P to the number of colonies of P was taken as the bacterial attachment rate (A_B_).

The benthic diatom attachment experiment was carried out to test the performance of the coating against benthic diatom attachment. The number of diatoms attached to the coating was characterized by measuring the final chlorophyll-a concentration on the surface of the glass slide sample. Three glass slide samples were selected for each coating for benthic diatom attachment experiments. In the first step, the sample was placed in a benthic diatom solution with a concentration of 0.3 μmol/mL and cultured in a light incubator for 48 h. In the second step, the sample was removed and rinsed with 30 mL of sterile seawater. In the third step, the rinsed sample was placed in a centrifuge tube containing 45 mL of 90% acetone solution, and 1 mL of 1% magnesium carbonate suspension was added to the centrifuge tube. In the fourth step, the centrifuge tube was put into a black bag and then placed in a dark environment at 8 °C for 24 h to extract chlorophyll. After 24 h, 15 mL of the solution was taken for centrifugation (4000 rpm, 10 min). Then, the supernatant was taken to measure the absorbance at wavelengths of 750 nm, 663 nm, 645 nm, and 630 nm. Finally, the chlorophyll-a concentration value was calculated by Equation (1) to characterize the attachment number of diatoms on the coating. To compare the antifouling performance more intuitively, the ratio of the chlorophyll-a concentration of CNTC/P and CNC/P to the chlorophyll-a concentration of P was taken as the benthic diatom attachment rate (A_D_).

Chlorophyll-a (mg/L) = 11.64 × (OD663) − 2.16 × (OD645) + 0.10 × (OD630)(1)

In the formula, OD663, OD645, and OD630 are the absorbance values at 663 nm, 645 nm, and 630 nm minus the absorbance value at 750 nm, respectively.

## 3. Results and Discussion

### 3.1. Microstructure and Property of the Composite Powders

#### 3.1.1. Phase Composition and Crystal Structure

**Figure 1:**
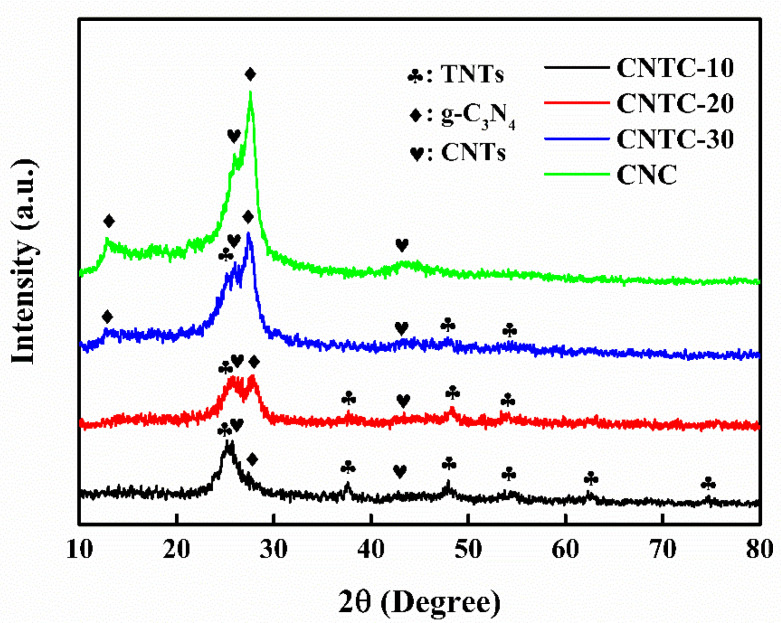
XRD patterns of CNTC and CNC.

The crystal structures of CNTC and CNC were analyzed using XRD, as shown in Figure 1. CNTC appeared with two characteristic peaks at 13.1° and 27.5°, corresponding to the in-plane stacking diffraction peaks of the (100) crystal plane of g-C_3_N_4_ and the interlayer stacking peaks of the graphite-like conjugated aromatic rings of the (002) crystal plane [27], The characteristic peaks at 25.2°, 37.8°, 48.0°, 55.0°, 62.7°, and 75.0° were anatase TiO_2_, corresponding to (101), (004), (200), (211), (204) and (215) crystal plane. A sharp peak appeared at 26.0° and a broad peak at 42.5°, corresponding to the (002) and (100) planes of CNTs, respectively. As the mass ratio of g-C_3_N_4_ in CNTC increased, the intensity of characteristic peaks of g-C_3_N_4_ increased. While the intensity of diffraction peaks for TNTs and CNTs decreases relatively. CNTC-10, CNTC-20, and CNTC-30 all appeared with characteristic peaks of g-C_3_N_4_, TNTs, and CNTs, and the intensity of diffraction peaks decreased and enhanced, indicating the successful recombination of CNTC. Characteristic peaks of g-C_3_N_4_ and CNTs appeared in CNC, indicating the successful recombination of CNC. In addition, no other characteristic peaks appeared in the XRD spectrum, which proved that there were no impurities in CNTC and CNC.

#### 3.1.2. Microstructure and Morphology

**Figure 2:**
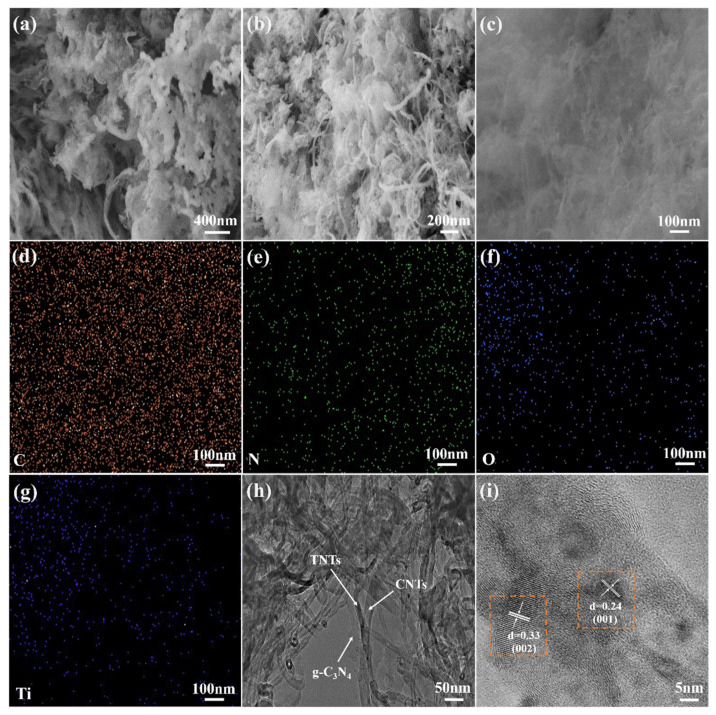
(**a**) SEM image of g-C3N4/TNTs; (**b**,**c**) SEM image of CNTC-20; (**d**–**g**) EDS image of CNTC-20; (**h**) TEM image of CNTC-20; (**i**) CNTC-20 of high-resolution TEM images.

To visually verify the successful recombination of g-C_3_N_4_/TNTs and CNTC, the recombination of g-C_3_N_4_/TNTs and CNTC-20 was observed and analyzed by SEM and TEM, and the element distribution was observed by EDS. In Figure 2a, a large amount of large-sized flake g-C_3_N_4_ can be seen, while few exposed TNTs can be seen. This is because, during the thermal polymerization process, g-C_3_N_4_ took TNTs as the skeleton and intertwined around the outer layer under the support of TNTs. From Figure 2b, it can be seen that g-C_3_N_4_ exhibited a thinner lamellar wrinkle shape. In addition, TNTs and CNTs exhibit an interwoven tubular shape. It indicated that during the ultrasonic and stirring dispersion in IPA, g-C_3_N_4_/TNTs and CNTs not only successfully combined with CNTs, but also exfoliated g-C_3_N_4_ into lamellae. After the g-C_3_N_4_/TNTs were combined with CNTs, the TNTs and CNTs were intertwined and become the skeleton of g-C_3_N_4_. In this way, the obtained g-C_3_N_4_ was looser and the specific surface area also increased. The EDS images were obtained in Figure 2c, as shown in Figure 2d–g. The distribution of C, N, O, and Ti elements can be seen, indicating that g-C_3_N_4_, TNTs, and CNTs were uniformly distributed in the CNTC composite powder. Figure 2h is the TEM image of CNTC-20, it can be seen more clearly that TNTs and CNTs were intertwined, and g-C_3_N_4_ was distributed on the surface of TNTs and CNTs. Figure 2i is a high-resolution TEM image of CNTC-20. It can be seen that g-C_3_N_4_, TNTs, and CNTs formed a heterojunction surface. In addition, lattice fringes can be seen, and the lattice fringe spacing is 0.33 nm corresponding to the (002) crystal plane of CNTs, and 0.24 nm corresponding to the (001) crystal plane of TNTs. The TEM test results further indicated that the CNTCs were successfully recombined, and a good heterointerface was formed between them.

#### 3.1.3. XPS Analysis

**Figure 3:**
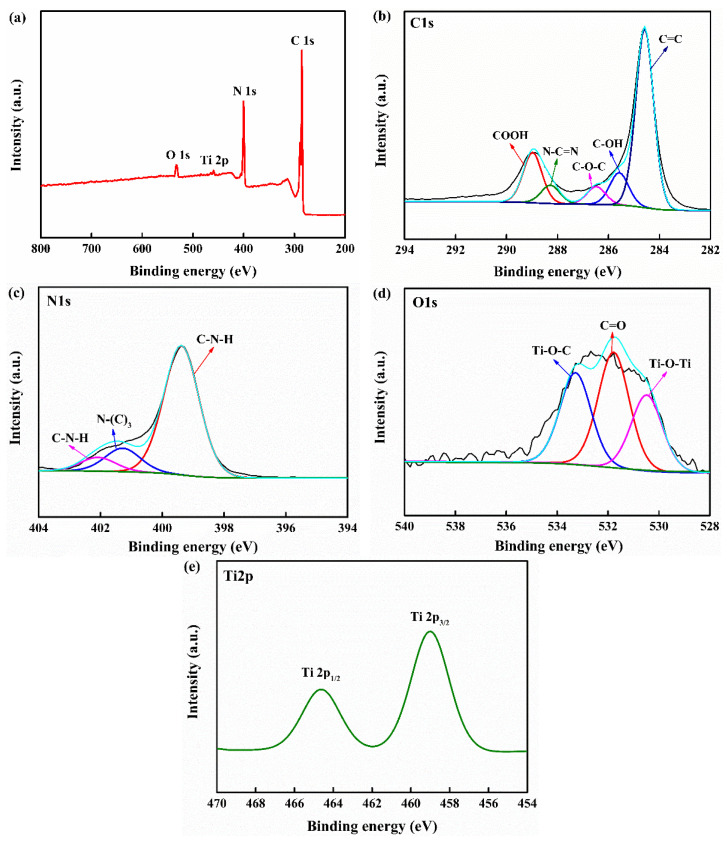
(**a**) XPS survey spectrum of CNTC-20; (**b**) C1s high-resolution XPS spectrum of CNTC-20; (**c**) N1s high-resolution XPS spectrum of CNTC-20; (**d**) O1s high-resolution XPS spectrum of CNTC-20; (**e**) Ti2p high-resolution XPS spectrum of CNTC-20.

The surface element composition and chemical bonds of CNTC were analyzed by XPS and the formation of its heterostructure was verified, as shown in Figure 3. Figure 3a is the survey spectrum of CNTC, which confirms the existence of C, N, O, and Ti elements in the CNTC composite powder. Figure 3b shows the high-resolution spectrum of the C1s of CNTC, where the peak at the binding energy of 284.5 eV corresponds to C=C. Peaks appeared at 285.5 eV and 286.4 eV, corresponding to the C-OH and C-O-C functional groups, respectively [28]. It can be judged that C-OH and C-O-C are functional groups generated after the composite, which proves that the CNTC composite powder formed a heterostructure. The peak at 288.2 eV corresponds to the sp^2^ hybridized c(N-C=N) of g-C_3_N_4_. The peak at 288.9 eV corresponds to the COOH functional group in CNTs [20]. Because most of the composite CNTs wound on the surface. As well, there was an interaction of the interface after composite, some of the peaks generated by N-C=N in g-C_3_N_4_ were masked. Figure 3c is a high-resolution spectrum of N1s of CNTC, in which three peaks are observed. 399.3 eV corresponds to sp^2^-hybridized C-N=C, and 401.2 eV and 402.0 eV correspond to the trinitrogen (N-(C)_3_) and two-coordinate nitrogen atoms (C-N-H) in the g-C_3_N_4_ heterocycle, respectively [29]. Figure 3d is the high-resolution spectrum of O1s of CNTC, with peaks at binding energies of 530.4 eV, 531.7 eV, and 533.2 eV, corresponding to Ti-O-Ti, C=O, and Ti-O-C bonds, respectively [28]. The formation of Ti-O-C indicated the formation of chemical bonds between CNTs and TNTs. The formation of chemical bonds between them could accelerate electron transfer, which helped to improve the photocatalytic performance. Figure 3e shows the high-resolution spectrum of Ti2p of CNTC, with peaks at 459.0 eV and 464.6 eV belonging to Ti2p_3/2_ and Ti2p_1/2_ in TNTs [30]. The successful composite of CNTC and the formation of heterostructure were further verified by the results of XPS. This result is consistent with the XRD and TEM test results.

#### 3.1.4. Photoluminescent and Photocatalytic Performance

**Figure 4:**
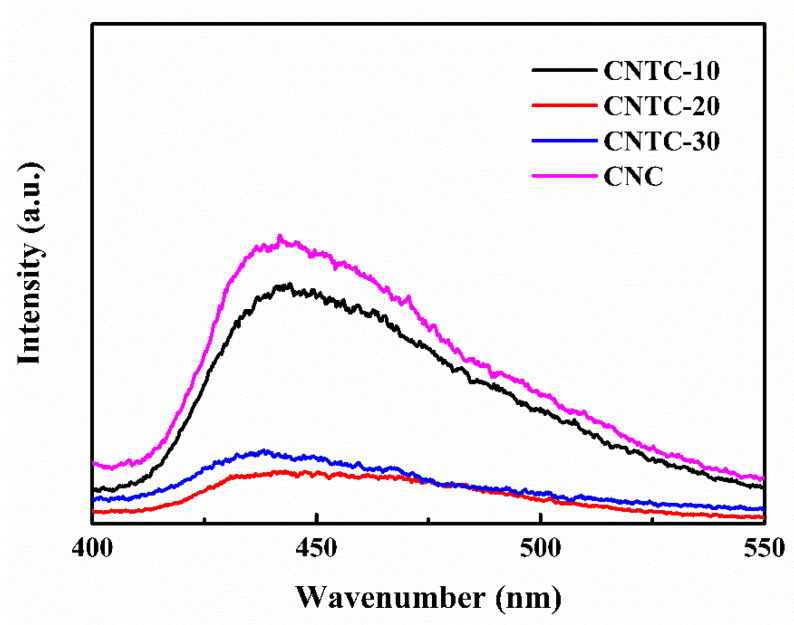
Photoluminescence spectra of CNTC and CNC.

The separation efficiency of photogenerated carriers is an essential factor affecting photocatalytic activity. The PL of CNTC and CNC was tested by a fluorescence spectrophotometer under an excitation light source with a wavelength of 365 nm, and the results are shown in Figure 4. In general, fluorescence intensity can be used to characterize the recombination rate of electron-hole pairs [31]. The stronger the fluorescence intensity is, the faster the recombination rate of electron-hole pairs increases. As the ratio of g-C_3_N_4_ in CNTC increased, the relative intensity of fluorescence first decreased and then increased. As well, the relative intensity of CNTC-20 was the smallest. Compared with CNC, the position of the emission peak (~440 nm) of CNTC did not change significantly but the intensity changed considerably. It can be seen that the luminescence intensity of CNTC obtained by recombining TNTs with g-C_3_N_4_ is lower than that of CNC, indicating that the recombination rate of electron-hole pairs was reduced after the recombination of TNTs and g-C_3_N_4_. The intensity difference mainly originates from the heterostructure formed in CNTC, which leads to a change in the electron transfer path and improves the separation of electron-hole pairs. In addition, TNTs are highly interconnected with CNTs under the joint action of CNTs. The CNTC has efficient charge transfer and reduces the loss caused by electron-hole recombination. But for CNC without TNTs, the emission peak is attributed to the photogenerated electron recombination of g-C_3_N_4_. Therefore, it can be judged that the photocatalytic performance of CNTC is better according to the recombination of electron-hole pairs.

### 3.2. Properties of the Coatings

#### 3.2.1. Surface Morphology and Roughness

**Figure 5:**
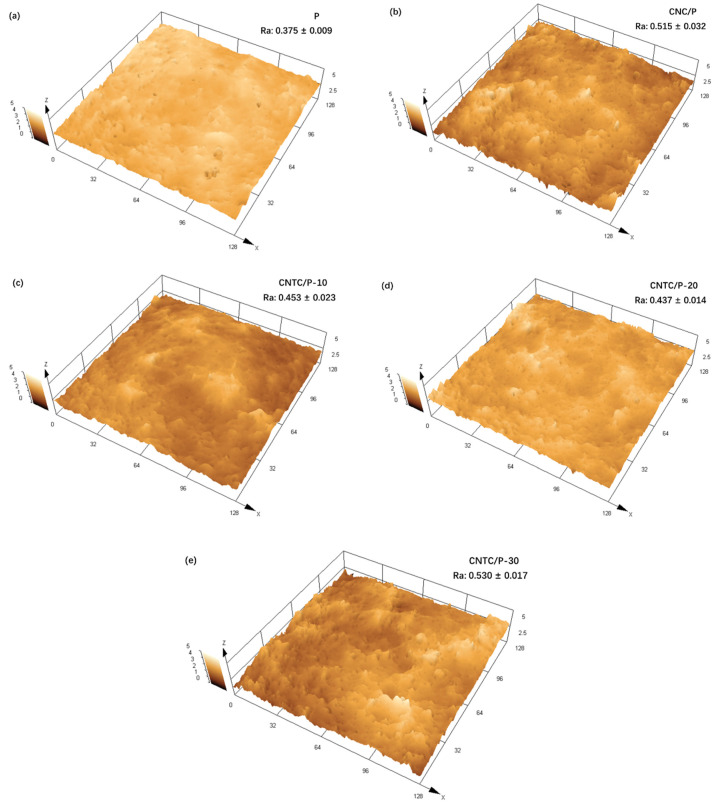
CLSM topography and roughness of the coatings (**a**) Coating P; (**b**) Coating CNC/P; (**c**) Coating CNTC/P-10; (**d**) Coating of CNTC/P-20; (**e**) Coating CNTC/P-30.

The surface properties of the coatings have an important impact on the attachment behavior of fouling organisms. The surface topography and roughness of the coatings were tested using CLSM, as shown in Figure 5. More irregular microscopic protrusions appeared on the surfaces of CNTC/P and CNC/P. It can also be seen that the addition of CNTC and CNC leads to an increase in roughness. This is due to the added CNTC and CNC, which contained many CNTs and TNTs with linear tubular structures. During the mixing process, they became entangled, resulting in increased roughness. Among them, CNTC/P-30 has the highest roughness due to the higher g-C_3_N_4_ content. g-C_3_N_4_ is a nano-powder with a large specific surface area, which is more likely to agglomerate inside the coating and leads to an increase in the roughness of the coating after agglomeration. In conclusion, although the addition of CNTC and CNC led to an increase in the roughness of CNTC/P and CNC/P, the roughness of all coatings was maintained at a low level.

#### 3.2.2. Wettability and Surface Energy

**Figure 6:**
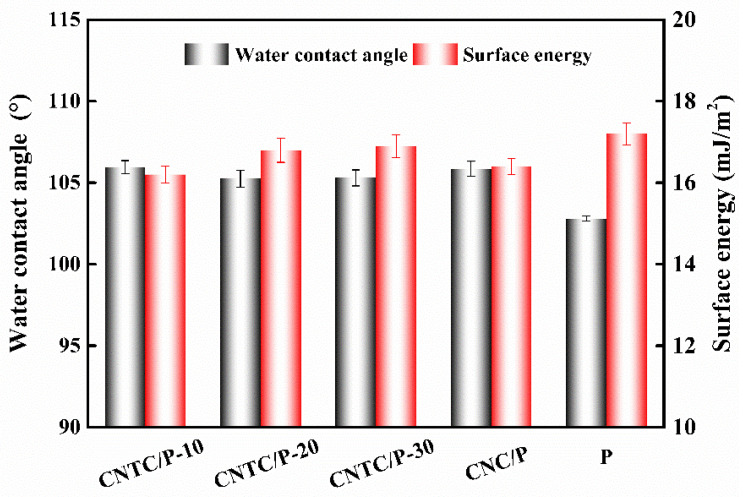
WCA and SE of studied coatings.

The measured WCA and SE of each coating are shown in Figure 6. The WCA of the coating with composite powder is about 105° and about 3° higher than that of the P. The WCA results are similar to those reported in the literature, and the addition of nano-powders can slightly improve the hydrophobicity of the coating [7,32]. This is because when the amount of nano-powder added is small, the PDMS in the coating can completely wrap the powder inside the coating. Therefore, the hydrophobicity of the coating mainly depends on the PDMS. On the other hand, the presence of nano-powders can cause the roughness of the coating to increase. According to the Cassie model theory, an increase in the roughness of a hydrophobic surface leads to an increase in the gas between the rough surface and the water droplets [33]. The contact area between the water droplet and the coating decreases, thereby increasing the WCA. Similarly, the increase of WCA and roughness leads to the decrease of coating SE, the SE of the coating CNTC and CNC is about 16 mJ/m^2^, both slightly smaller than that of the coating P; the SE of all studied coatings is much smaller than that of pure PDMS 25 mJ/m^2^ [34].

#### 3.2.3. Antifouling Performance

**Figure 7:**
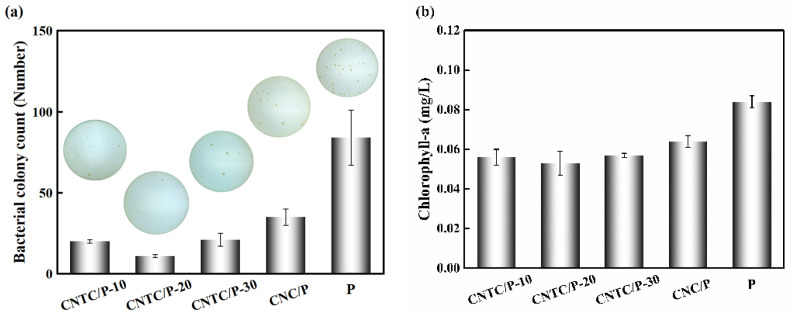
(**a**) Bacterial colony attached on studied coatings; (**b**) Chlorophyll-a concentration of the diatom attached on studied coatings.

A bacterial attachment test is an effective method to analyze the antifouling performance of coatings. To reflect the antifouling performance of the coating in the ocean, fresh seawater rich in bacteria was selected for the experiment. After culturing for 48 h, the results of bacterial attachment experiments are shown in Figure 7. It can be seen intuitively that the number of colonies of CNTC/P and CNC/P is significantly lower than that of P. The number of colonies of P is 84 and more than that of CNTC/P and CNC/P. This is due to the addition of CNTC and CNC, which made the coatings function as photocatalytic sterilization. The CNTC and CNC in the coatings underwent a photocatalytic reaction under light irradiation to generate ROS with strong oxidative power to kill bacterial microorganisms. The bacterial attachment rates (A_B_) were 23.8%, 13.1%, 25.0% and 41.7%. In contrast, the antifouling effect of adding CNTC is better than adding CNC.

In the study of improving the performance of silicone coatings, Soleimani et al. added reduced graphene oxide/Ag nanocomposite and multi-walled carbon nanotubes into PDMS, which improved the antibacterial properties of the coating by 43.9% [35]. Yang et al. added CNTs and TiO_2_ to PDMS to investigate their antifouling properties, and the results showed that the biofilm attachment amount was reduced by 50% after 28 days [36]. In the two antifouling strategies, the antibacterial properties of nano-Ag and TiO_2_ are mainly relied on. Nano-Ag is a heavy metal and will do harm to the environment if released into the ocean. The photocatalytic activity of TiO_2_ under visible light is very low, and its antibacterial properties are poor. In contrast, CNTC is a more environmentally friendly powder and has better antibacterial properties under visible light. Therefore, it is an effective method to improve the antibacterial properties of PDMS by nanocomposite powder. As well, this photocatalytic nanocomposite powder with visible light response shows good antibacterial properties.

The ability to resist algae attachment was analyzed by the benthic diatom attachment experiment. By extracting the Chlorophyll-a on the coating, the attached benthic diatoms on the coating were characterized. The experimental results are shown in Figure 7b, the chlorophyll concentration of CNTC/P and CNC/P is lower than that of P (0.084 mg/L). The chlorophyll concentration of CNTC/P is lower than that of CNC/P. The benthic diatom attachment rates (A_D_) were 66.7%, 63.1%, 67.9% and 76.2%, respectively. There was no significant difference in A_D_ between CNTC/P-10, CNTC/P-20, and CNTC/P-30, indicating that their ability to inhibit the attachment of benthic diatoms was similar. The addition of CNTC and CNC has a certain inhibitory effect on the attachment of benthic diatoms. Still, the effect of inhibiting its attachment is not as obvious as that of inhibiting bacterial attachment. In other studies, L. H. Yang et al. composited polyacrylic acid (PAA)/TiO_2_ with PDMS to construct a biomimetic shark skin structure through self-assembly technology, and the inhibition rate of the coating on diatoms reached 66% and 78% [37]. This biomimetic structure has better resistance to the attachment of benthic diatoms. The bionic structure has an all-weather antifouling effect while the antifouling effect of the photocatalytic composite powder requires an external light source. In addition, benthic diatoms settle at night, but the coatings have poor antifouling properties at night. Therefore, the coating inhibiting the adhesion of benthic diatoms is related to the antifouling mechanism and the adhesion mechanism of benthic diatoms. How to prolong the photocatalytic activity time is also a direction for future research.

### 3.3. Discussion on Antifouling Mechanism

**Figure 8:**
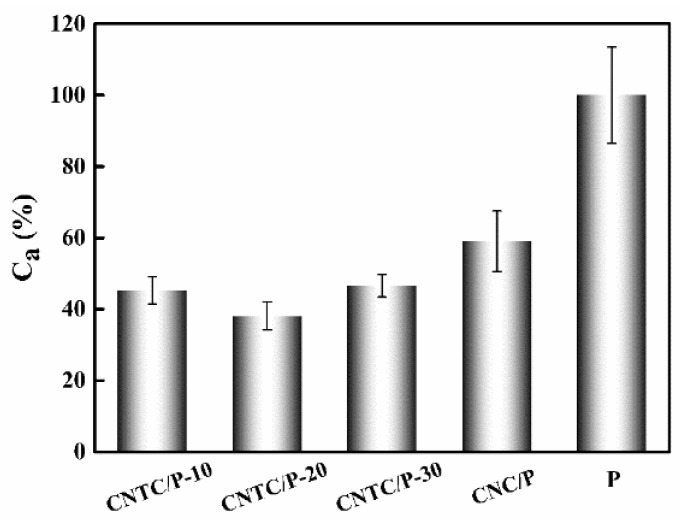
Comprehensive fouling adhesion rate (C_a_) of studied coatings.

To better evaluate the antifouling performance of the coating, the average value of the sum of A_B_ and A_D_ of CNTC/P and CNC/P was taken as the comprehensive fouling adhesion rate (C_a_) and the C_a_ of P was 100%. The C_a_ of CNTC/P and CNC/P is shown in Figure 8. The comprehensive fouling adhesion rates of CNTC/P-10, CNTC/P-20, CNTC/P-30 and CNC/P were 45.3%, 38.1%, 46.5% and 59.0%, respectively. CNTC and CNC significantly improved the comprehensive antifouling performance of PDMS coating, among which CNTC/P-20 was the best.

Under light irradiation with energy higher than its band gap, photocatalytic materials generated a series of ROS including OH, O_2_^−^, h^+^, e^−^, etc [38]. ROS could inactivate bacteria and microalgae by destroying their cell walls [39]. The improvement of the comprehensive antifouling performance of the coatings was due to the addition of CNTC and CNC. This made the coatings have photocatalytic activity under the irradiation of visible light and a redox reaction occurred to generate ROS. The TEM test results showed that g-C_3_N_4_, TNTs, and CNTs formed an obvious heterogeneous interface. This promoted the interfacial electron transfers so that the recombination of photogenerated carriers was suppressed, thereby increasing the photogenerated electron-hole pairs that undergo redox reactions at the surface. The XPS analysis results showed that the generation of C-O bonds was due to the formation of heterostructures. A heterointerface was formed after recombination, thereby enhancing the photocatalytic activity. The CNTs in CNTC and CNC also facilitated the transfer of photogenerated electrons. Therefore, with their excellent photocatalytic activity, CNTC and CNC greatly enhanced the antifouling performance of the coatings. The comprehensive antifouling performance of CNTC/P was better than that of CNC/P. The PL test results showed that the luminescence intensity of CNTC was significantly lower than that of CNC, which proved that the photogenerated electron-hole recombination rate of g-C_3_N_4_ was faster. Combining g-C_3_N_4_ with TNTs effectively reduced the recombination rate of photogenerated electrons and holes, thereby significantly improving the photocatalytic activity. For CNTC/P, Ca first decreased and then increased with the increase of g-C_3_N_4_ in CNTC, indicating that the recombination efficiency of g-C_3_N_4_ and TNTs was related to the content. This may be because the g-C_3_N_4_ in CNTC-20 sufficiently utilized the larger specific surface area of TNTs to provide more active sites for photocatalytic reactions. If the content of g-C_3_N_4_ was too small, the surface of TNTs could not be fully utilized. However, the excessive content of g-C_3_N_4_ led to the extreme thickness of g-C_3_N_4_ covering the surface of TNTs, thereby reducing the light utilization rate. The number of CNTC heterointerfaces may also have a particular effect on photocatalytic activity [30,40]. Free electrons were transferred from the conduction band of g-C_3_N_4_ to TNTs through the heterointerface. This effectively reduced the electron-hole recombination rate, thereby producing more active species to kill bacteria and diatoms. However, the electron-hole recombination rate of CNTC-30 was lower than that of CNTC-10, while the C_a_ increased by 1.2%. It may be due to the more significant roughness of CNTC/P-30. There were more stress points provided so that the attachment of bacteria and diatoms increased. In addition, the efficiency of the coating to inhibit the attachment of benthic diatoms is also affected by the mechanism of the physiological activity of benthic diatoms. Benthic diatoms have the characteristic of nocturnal sedimentation, which causes them to attach to the coating at night [41]. For CNTC and CNC, there is no light source at night and the photocatalytic reaction cannot proceed, resulting in the coating relying solely on the low SE properties of the silicone coating to inhibit the attachment of benthic diatoms. When there is a light source in the daytime, the g-C_3_N_4_ and TNTs in the composite powder have photocatalytic activity. The generated active substances make the partially attached benthic diatoms detach from the coating. Therefore, CNTC/P and CNC/P can effectively inhibit the adhesion of benthic diatoms, but the inhibitory effect is not as apparent as that of inhibiting the adhesion of bacteria.

The surface properties of the coatings are essential factors affecting the attachment of bacteria and diatoms. From the analysis of the surface properties of the coating, the addition of CNTC and CNC to the PDMS coating improved the hydrophobicity of the coatings. For the fouling release antifouling coating, the improvement of hydrophobicity has a particular effect on improving the antifouling performance. The SE of CNTC/P and CNC/P decreases slightly compared to P. The coating relies on its low SE, making it difficult for fouling organisms to attach to its surface. For PDMS composite antifouling coatings, low SE is the primary source of fouling release performance [42]. Although the addition of CNTC and CNC increases the roughness of the coating with the maximum value of 0.53 μm, it remains at a low level. The smooth surface benefits from the flexible segments of PDMS, making the coating less undulating. Because of the smooth surface of the CNTC/P, the sticky substances secreted by the fouling organisms cannot adhere firmly to the surface [43]. Therefore, benefiting from the low SE and roughness of CNTC/P, the coating can have excellent antifouling properties. It is worth noting that despite the low SE and low roughness of P, its ability to inhibit the attachment of bacteria and benthic diatoms is worse due to its absence of CNTC and CNC. It showed that adding composite powder significantly improves the antifouling performance of the coating.

## 4. Conclusions

(1) The recombination of g-C_3_N_4,_ TNTs, and CNTs reduced the recombination rate of photogenerated carriers in g-C_3_N_4_. Among several composite powders, CNTC-20 had the lowest recombination rate of photogenerated carriers.
(2) When the composite powder was added to PDMS antifouling coating, the roughness and WCA of the coating increased, while the SE decreased.
(3) The addition of CNTC and CNC improved the antibacterial and benthic diatom adhesion performance of the coating. The antifouling effect of CNTC/P was better than that of CNC/P, indicating that the photocatalytic activity of g-C_3_N_4_ modified by TNTs was improved, which improved the antifouling performance of the coating. Among the studied coatings, CNTC/P-20 had the best antifouling performance with a bacterial attachment rate of only 13.1% and a benthic diatom attachment rate of 63.1%.
(4) It is feasible to enhance the photocatalytic activity of g-C_3_N_4_ by constructing a heterostructure to improve the antifouling performance of the coating. The introduction of the photocatalytic composite powder enables the coating to have self-cleaning properties. Meanwhile, the composite coating has lower SE and roughness, so it is difficult for fouling organisms to adhere firmly to its surface. Therefore, this strategy of combining photocatalytic materials with silicone antifouling coatings is an effective way to improve the antifouling performance of silicone coatings.

## Data Availability

Not applicable.
